# Association of dietary niacin intake with all-cause mortality in chronic kidney disease: A retrospective cohort study of NHANES

**DOI:** 10.1371/journal.pone.0313398

**Published:** 2025-02-07

**Authors:** Meijuan Xiang, Jianyun Peng, Zhihong Gui, Ju Jin, Jinling Meng

**Affiliations:** 1 Department of Nephrology, The Sixth Affiliated Hospital of Wenzhou Medical University, Lishui People’s Hospital, Lishui, P.R. China; 2 Zhejiang Chinese Medical University, Hangzhou City, Zhejiang Province, P.R. China; Boston University Chobanian & Avedisian School of Medicine, UNITED STATES OF AMERICA

## Abstract

**Background:**

Dietary niacin intake has a positive influence on several chronic diseases, while the impact of dietary niacin intake on prognosis in chronic kidney disease (CKD) remains unknown. The study would explore the association between dietary niacin intake and all-cause mortality in CKD patients.

**Methods:**

Data about 4,659 CKD patients in this retrospective cohort study were obtained from the National Health and Nutrition Examinations Survey (NHANES). Dietary niacin intake data were acquired based on the 24-hour dietary recall interviews. Weighted univariate Cox regression models were utilized to select potential covariates. The association of dietary niacin intake with all-cause mortality was explored using weighted univariate and multivariate Cox regression models. The results were presented as hazard ratios (HRs) with 95% confidence intervals (CIs).

**Results:**

In total, 4,659 CKD patients were included in the study. The mean age of patients was 58.03 (0.42) years old, and 2,502 (58.45%) were female. During a mean follow-up of 73.92 (1.14) months, 1,325 (28.44%) CKD patients died. Compared to CKD patients with lower niacin intake ≤19mg, those with niacin intake >33 mg were associated with lower all-cause mortality risk (HR = 0.79, 95%CI: 0.64–0.98). The association also found in subgroups of age ≥65 years old (HR = 0.68, 95%CI: 0.53–0.88), males (HR = 0.68, 95%CI: 0.51–0.92), BMI <25 kg/m^2^ (HR = 0.63, 95%CI: 0.39–0.99), smoking (HR = 0.68, 95%CI: 0.49–0.94), dyslipidemia (HR = 0.71, 95%CI: 0.56–0.91), and non-hyperphosphatemia (HR = 0.73, 95%CI: 0.58–0.91).

**Conclusion:**

Adequate dietary niacin intake was related to lower odds of all-cause mortality in CKD patients. Niacin supplements may have potential benefits for prognosis in CKD patients.

## Introduction

Chronic kidney disease (CKD) is a global health concern, characterized by the progressive decline in renal function over time [1]. CKD carries a huge disease burden, with a worldwide prevalence of 9.1% and an annual mortality of 1.2 million individuals [2]. Accordingly, identifying modifiable risk factors for mortality in CKD patients is of critical significance.

Nutrition plays a vital role in both the management and prognosis of CKD patients [1, 3]. Niacin, also known as vitamin B3, is an essential nutrient with diverse physiological functions, including involvement in energy metabolism, cellular signaling, and lipid metabolism [4]. Previous research has shown that niacin supplementation may have beneficial effects on lipid profiles and cardiovascular outcomes in the general population [5–7]. Remarkably, niacin possesses the ability to raise high-density lipoprotein cholesterol (HDL-C), lower low-density lipoprotein cholesterol (LDL-C), and reduce triglyceride (TG) levels [8]. These lipid-modifying effects could be potentially beneficial for CKD patients, given their heightened risk of cardiovascular disease (CVD). Additionally, niacin could improve dyslipidemia, limit phosphorus absorption, lower serum phosphorus levels, and maintain glomerular filtration rate in CKD patients, therefore slowing disease progression and improving prognosis [9–12]. Furthermore, niacin possesses anti-inflammatory and antioxidant properties, which are especially pertinent for individuals with CKD, given the critical role of inflammation and oxidative stress in the disease’s pathophysiology [1, 13].

Nevertheless, the relationship between niacin intake and all-cause mortality in CKD patients remains unclear. This study aimed to investigate the association of niacin intake with all-cause mortality in CKD patients. Considering the substantial relevance of this association for the nutritional management of CKD patients, it could significantly influence dietary recommendations and interventions aimed at enhancing prognosis within this population.

## Methods

### Study design and participants

The National Health and Nutrition Examinations Survey (NHANES), with a nationally representative sample, was conducted to assess the health and nutritional status of the United States population. The survey combined interviews and physical examinations. The requirement of ethical approval for this study was waived by the Institutional Review Board of the Lishui People’s Hospital, because the data was accessed from NHANES (a publicly available database).

In the present retrospective cohort study, data about CKD patients were extracted from 6 cycles (2007–2008, 2009–2010, 2011–2012, 2013–2014, 2015–2016, and 2017–2018) of NHANES. CKD was diagnosed with an estimated glomerular filtration rate (eGFR) <60 ml/min/1.73m^2^ or urine albumin-to-creatinine ratio ≥30 mg/g [14]. The included criteria were as follows: (1) age ≥18 years old, (2) with data on dietary niacin intake, and (3) with survival data. CKD patients would be excluded of those missing data on important covariates.

### Dietary niacin intake assessment

Dietary niacin intake data were acquired utilizing the dietary interview component, which assessed the energy, nutrients, and other dietary components intake from consumed food and beverages [15]. CKD patients participated in two 24-hour dietary recall interviews: the first in person at the Mobile Examination Center, and the second via telephone 3 to 10 days later. Supplemental niacin intake data comprised responses to inquiries regarding the consumption of dietary supplements within the past 24 hours. Niacin intake categories were defined based on participants’ dietary niacin intakes and divided into three groups. Due to the substantial lack of supplemental niacin data during the second-day recall, the dietary day-one sample weight was applied for weighting purposes.

### Covariates

Covariates included were age, gender, race, marital status, smoking, hemoglobin, phosphorus, white blood cell (WBC), diabetes, hypertension, dyslipidemia, and CVD. The race of CKD patients was categorized as non-Hispanic white, non-Hispanic black, and others. The marital status was classified into married, never married, and others. The hematologic and biochemical tests were measured in the laboratory at the Center for Disease Control and Prevention. Diabetes was diagnosed in those who met any of the conditions: (1) Hemoglobin A1C ≥6.5%, (2) fasting glucose ≥126 mg/dL, (3) serum glucose at 2 hours following a 75 g glucose load ≥200 mg/dL, (4) self-reported physician diagnosis of diabetes, and (5) use of insulin or other diabetes medication [16, 17]. Self-reported diabetes was determined of patients who answered “yes” to the question “Doctor told you have diabetes”. Hypertension was diagnosed with any of the following: (1) systolic blood pressure ≥130 mm/Hg and/or diastolic blood pressure ≥80 mm/Hg [18], (2) self-reported diagnosis of hypertension, (3) use of hypotensive medication. Self-reported hypertension was determined with a positive response to the question “Ever told you had high blood pressure”. Dyslipidemia should be diagnosed of patients who meet any of the following criteria: (1) total cholesterol (TC) ≥200 mg/dL or TG ≥150 mg/dL or LDL-C ≥130 mg/dL or HDL-C ≤40 mg/dL, (2) self-reported dyslipidemia, (3) self-reported hypercholesterolemia, or undergoing lipid-lowering therapy [19]. CVD was determined by a standardized medical status questionnaire with the question: Has a doctor or other health professional ever told you that you had angina or heart failure/coronary heart disease/strike/congestive heart failure? Patients with a positive response to the above questions were considered to have CVD [20]. Patients who took cardiovascular medication were also considered as CVD.

### Mortality and follow-up

Mortality information was obtained from linked data provided by the Centers for Disease Control and Prevention. All-cause mortality was determined using the International Classification of Diseases, 10th Revision (ICD-10) [21]. The NCHS has linked data with death certificate records from the National Death Index (NDI) (https://www.cdc.gov/nchs/datalinkage/mortality-public.htm). The follow-up period was calculated from the date of examination to the date of death or December 31, 2019.

### Statistical analysis

To guarantee an accurate population representation, the proper NHANES sampling weights (SDMVPSU, SDMVPTRA) were performed based on guidelines from the Centers for Disease Control and Prevention. Continuous data were expressed as means and standard errors, and t-tests were used to compare between the two groups. Categorical data were presented as counts and proportions, chi-square tests were utilized for group comparison. Weighted univariate Cox regression models were utilized to select potential covariates. Covariates were adjusted for age, gender, race, marital status, smoke, hemoglobin, phosphorus, WBC, diabetes, hypertension, dyslipidemia, and CVD. Restricted cubic spline (RCS) was conducted to examine the relationship between niacin intake and all-cause mortality risk among patients with CKD. The association of dietary niacin intake and all-cause mortality in CKD patients was investigated using weighted univariate and multivariate Cox regression models. Results were expressed as hazard ratios (HRs) and 95% confidence intervals (CIs). The association was further investigated among subgroups on age, gender, body mass index (BMI), smoking, and dyslipidemia. Information about missing values were presented in S1 and S2 Tables. The likelihood ratio test was used to calculate the *P* value for interactions. Python 3.9 was utilized for data cleaning and dealing with missing data, and SAS 9.4 (SAS Institute Inc., Cary, NC, USA) was performed for all statistical analyses. A two-sided *P*<0.05 was considered statistical significance.

## Results

### Characteristics of CKD patients

**Fig 1:**
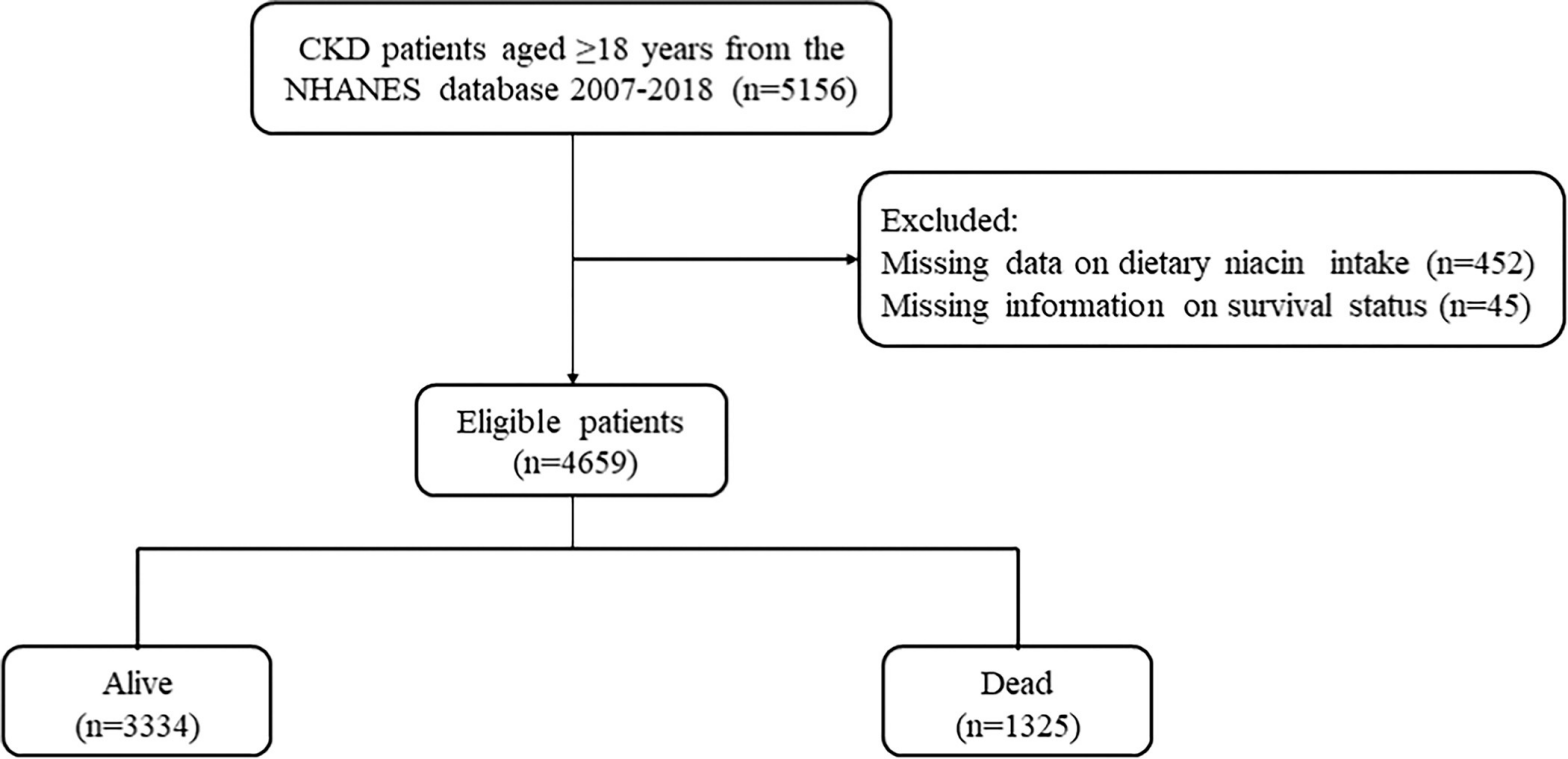
The flow chart of CKD patients selection. After screening, a total number of 4659 patients were included.

5,156 CKD patients were included in six consecutive NHANES 2-year cycles (2007–2018). CKD patients were excluded of those who did not have niacin intake data (n = 452) or with complete survival data (n = 45). In total, 4,659 CKD patients were included in the final analysis. The flowchart of the patient selection was shown in Fig 1. The mean age of patients was 58.03 (0.42) years old, 2,502 (58.45%) were female, and 1,993 (64.57%) were non-Hispanic white patients. During a mean follow-up of 73.92 (1.14) months, 1,325 (28.44%) CKD patients died. The average dietary niacin intake was 34.89 (1.41) mg/day. Table 1 shows the characteristics of included CKD patients. Statistical differences were found in age, gender, race, education level, marital status, smoking, drinking, physical activity, total energy, protein intake, diabetes, hypertension, dyslipidemia, CVD, cancer, WBC, hemoglobin, phosphorus, hyperuricemia, and angiotensin-converting enzyme inhibitor (ACEI)/ angiotensin II receptor blocker (ARB) between two groups.

**Table 1:** Characteristics of CKD patients.

Variables	Total (n = 4659)	All-cause mortality		*P*
		No (n = 3334)	Yes (n = 1325)	
Age, years, Mean (S.E)	58.03 (0.42)	54.46 (0.48)	69.24 (0.57)	<0.001*
Age, Mean (S.E)				<0.001^#^
<65	2329 (54.59)	1998 (62.60)	331 (29.42)	
≥65	2330 (45.41)	1336 (37.40)	994 (70.58)	
Gender, n (%)				<0.001^#^
Male	2157 (41.55)	1453 (39.98)	704 (46.46)	
Female	2502 (58.45)	1881 (60.02)	621 (53.54)	
Race, n (%)				<0.001^#^
Non-Hispanic White	1993 (64.57)	1245 (61.62)	748 (73.86)	
Non-Hispanic Black	1116 (13.59)	817 (13.94)	299 (12.49)	
Others	1550 (21.84)	1272 (24.44)	278 (13.64)	
Education level, n (%)				<0.001^#^
Less than high school	1488 (22.77)	976 (20.09)	512 (31.18)	
High school	1136 (26.70)	800 (26.37)	336 (27.76)	
College and above	2035 (50.53)	1558 (53.54)	477 (41.06)	
Marital status, n (%)				<0.001^#^
Married	2228 (49.87)	1630 (51.04)	598 (46.18)	
Never married	553 (13.10)	457 (14.65)	96 (8.22)	
Others	1878 (37.03)	1247 (34.30)	631 (45.60)	
PIR, n (%)				0.836^#^
<1.0	1172 (19.29)	857 (19.21)	315 (19.53)	
≥1.0	3487 (80.71)	2477 (80.79)	1010 (80.47)	
Smoking, n (%)				<0.001^#^
No	2377 (51.12)	1826 (54.36)	551 (40.95)	
Yes	2282 (48.88)	1508 (45.64)	774 (59.05)	
Drinking, n (%)				0.001^#^
No	1684 (32.95)	1186 (31.34)	498 (38.02)	
Yes	2975 (67.05)	2148 (68.66)	827 (61.98)	
Physical activity, n (%)				<0.001^#^
<450 met*minutes/week	521 (11.71)	386 (12.26)	135 (9.99)	
≥450 met*minutes/week	2224 (51.39)	1810 (57.26)	414 (32.92)	
Unknown	1914 (36.90)	1138 (30.48)	776 (57.09)	
Total energy, kcal, Mean (S.E)	1908.91 (19.75)	1955.55 (24.82)	1762.25 (35.44)	<0.001*
Protein intake, gm, Mean (S.E)	73.40 (0.88)	74.93 (1.01)	68.58 (1.81)	0.003*
Diabetes, n (%)				<0.001^#^
No	2688 (63.50)	2045 (67.19)	643 (51.92)	
Yes	1971 (36.50)	1289 (32.81)	682 (48.08)	
Hypertension, n (%)				<0.001^#^
No	1146 (29.86)	1007 (35.80)	139 (11.15)	
Yes	3513 (70.14)	2327 (64.20)	1186 (88.85)	
Dyslipidemia, n (%)				<0.001^#^
No	911 (21.10)	717 (23.43)	194 (13.79)	
Yes	3748 (78.90)	2617 (76.57)	1131 (86.21)	
CVD, n (%)				<0.001^#^
No	2586 (60.61)	2099 (67.47)	487 (39.06)	
Yes	2073 (39.39)	1235 (32.53)	838 (60.94)	
Depression, n (%)				0.050^#^
No	4119 (89.52)	2963 (90.13)	1156 (87.60)	
Yes	540 (10.48)	371 (9.87)	169 (12.40)	
Cancer, n (%)				<0.001^#^
No	3900 (82.76)	2897 (85.67)	1003 (73.64)	
Yes	759 (17.24)	437 (14.33)	322 (26.36)	
BMI, kg/m^2^, Mean (S.E)	30.32 (0.19)	30.44 (0.22)	29.98 (0.28)	0.188*
BMI, n(%)				0.138^#^
<25 kg/m^2^	1144 (25.63)	777 (24.99)	367 (27.69)	
≥25 kg/m^2^	3406 (74.37)	2512 (75.01)	894 (72.31)	
WBC, 1000 cell/uL, Mean (S.E)	7.69 (0.06)	7.63 (0.05)	7.90 (0.13)	0.040*
Hemoglobin, g/dL, Mean (S.E)	13.75 (0.05)	13.85 (0.05)	13.42 (0.10)	<0.001*
Phosphorus, Mean (S.E)	3.76 (0.01)	3.73 (0.01)	3.86 (0.03)	<0.001*
Hyperuricemia, n (%)				<0.001^#^
No	2996 (64.35)	2252 (67.20)	744 (55.38)	
Yes	1663 (35.65)	1082 (32.80)	581 (44.62)	
ACEI/ARB, n (%)				<0.001^#^
No	3441 (75.76)	2521 (77.61)	920 (69.95)	
Yes	1218 (24.24)	813 (22.39)	405 (30.05)	
Nephrotoxic drugs, n (%)				0.408^#^
No	4337 (93.56)	3094 (93.35)	1243 (94.22)	
Yes	322 (6.44)	240 (6.65)	82 (5.78)	
Follow-up time, Mean (S.E)	73.92 (1.14)	77.69 (1.36)	62.06 (2.52)	<0.001*
Niacin intake, mg, Mean (S.E)	34.89 (1.41)	34.77 (1.61)	35.25 (2.64)	0.875*
Niacin intake, n (%)				0.362^#^
≤19mg	1720 (33.29)	1215 (32.60)	505 (35.48)	
19-33mg	1505 (33.40)	1070 (33.64)	435 (32.64)	
>33mg	1434 (33.31)	1049 (33.77)	385 (31.88)	

* t tests, # Chi-square tests, S.E: standard error.

CKD: chronic kidney disease, PIR: poverty income ratio, CVD: cardiovascular disease, BMI: body mass index, WBC: white blood cell, ACEI: angiotensin-converting enzyme inhibitors, ARB: angiotensin II receptor blockers.

### Associations of dietary niacin intake with all-cause mortality in CKD patients

**Fig 2:**
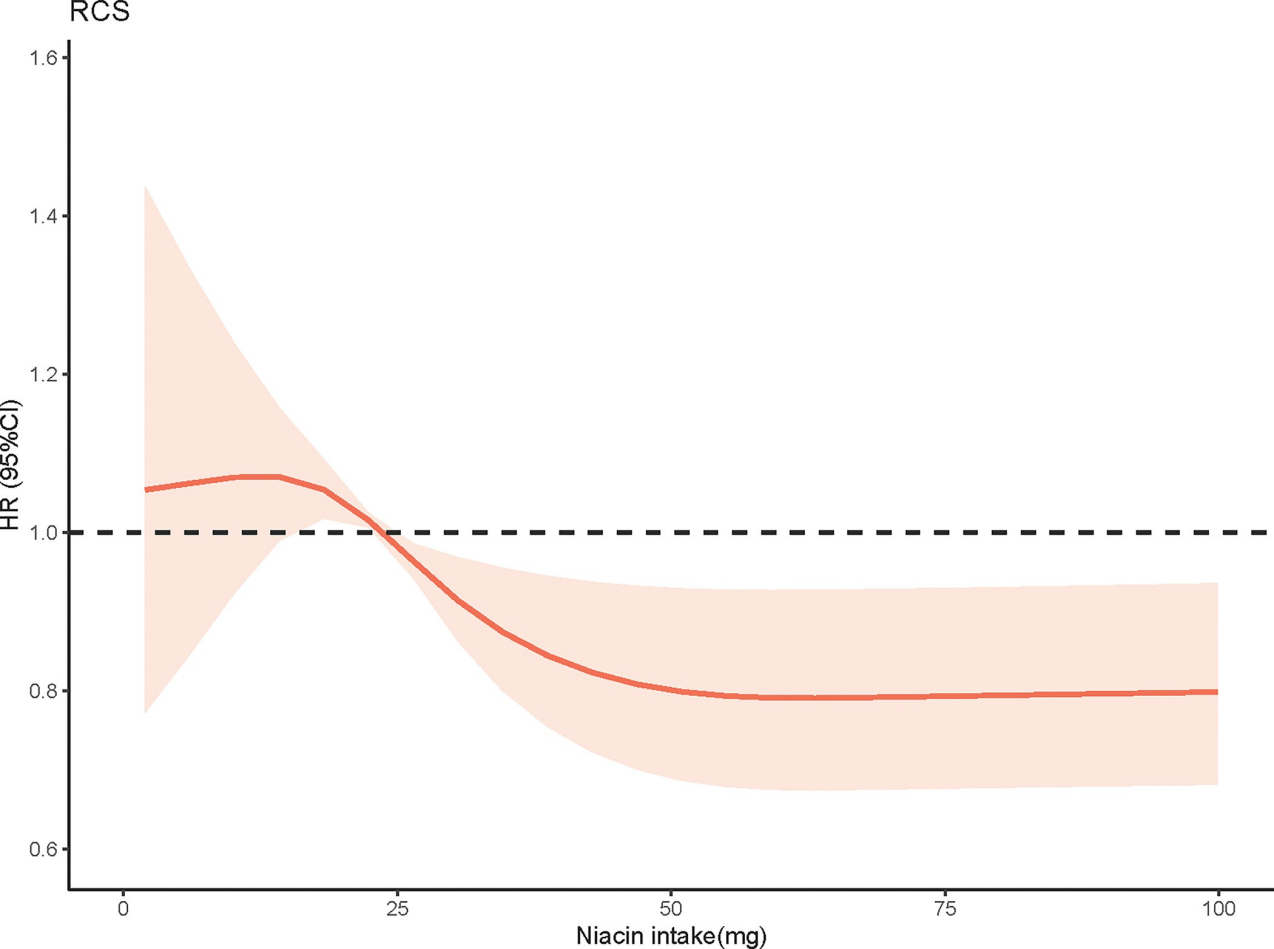
RCS analyses between niacin intake and all-cause mortality.

The relationship between dietary niacin intake and all-cause mortality was presented in Table 2. After adjusting for demographical covariates in model 1, higher niacin intake was related to lower all-cause mortality risk in CKD patients (HR = 0.80, 95%CI: 0.66–0.96). In adjusted model 2, model 3 and model 4, compared to patients with niacin intake ≤19mg, those with higher niacin intake (>33 mg) were associated with lower all-cause mortality risk (model 2: HR = 0.80, 95%CI: 0.67–0.96; model 3: HR = 0.74, 95%CI: 0.59–0.92; model 4: HR = 0.74, 95%CI: 0.59–0.93). Fig 2 indicates a nonlinear relationship between niacin intake and all-cause mortality (*P* for nonlinear = 0.004).

**Table 2:** The associations of niacin intake with all-cause mortality in CKD patients.

Variables	Model 1		Model 2		Model 3		Model 4	
	HR (95%CI)	*P*	HR (95%CI)	*P*	HR (95%CI)	*P*	HR (95%CI)	*P*
Niacin intake	0.90 (0.82–0.99)	0.035	0.92 (0.83–1.01)	0.073	0.90 (0.79–1.01)	0.070	0.90 (0.79–1.01)	0.074
Niacin intake								
≤19mg	Ref		Ref		Ref		Ref	
19-33mg	0.96 (0.80–1.14)	0.622	0.97 (0.82–1.14)	0.673	0.97 (0.81–1.16)	0.713	0.97 (0.80–1.17)	0.751
>33mg	0.80 (0.66–0.96)	0.018	0.80 (0.67–0.96)	0.018	0.74 (0.59–0.92)	0.008	0.74 (0.59–0.93)	0.010

HR: hazards ratio; CI: confidence interval; Ref: reference.

CKD: chronic kidney disease.

Model 1: adjusting age, gender, and race.

Model 2: adjusting age, gender, race, marital status, smoking, hemoglobin, phosphorus, and white blood cell.

Model 3: adjusting age, gender, race, marital status, smoking, hemoglobin, phosphorus, white blood cell, diabetes, hypertension, dyslipidemia, cardiovascular disease, albumin creatinine ratio, eGFR, survey years, and total energy intake.

Model 4: adjusting age, gender, race, marital status, smoking, hemoglobin, phosphorus, white blood cell, diabetes, hypertension, dyslipidemia, cardiovascular disease, albumin creatinine ratio, eGFR, survey years, total energy intake, TC, BMI, SBP, DBP, and antihypertensive medications.

### Dietary niacin intake and mortality within subgroups of age, gender, BMI, smoking, and dyslipidemia

Table 3 presents the association between niacin intake and all-cause mortality which is stratified by age, gender, BMI, smoking, dyslipidemia, and hyperphosphatemia. Higher niacin intake was associated with increased all-cause mortality risk in those age ≥65 years old (HR = 0.68, 95%CI: 0.53–0.88), males (HR = 0.68, 95%CI: 0.51–0.92), BMI <25 kg/m^2^ (HR = 0.70, 95%CI: 0.39–0.99), smoking (HR = 0.68, 95%CI: 0.49–0.94), dyslipidemia (HR = 0.71, 95%CI: 0.56–0.91), and non-hyperphosphatemia (HR = 0.73, 95%CI: 0.58–0.91). There was an interaction between niacin intake and dyslipidemia for the association between niacin intake and all-cause mortality (*P* for interaction = 0.030).

**Table 3:** The associations between niacin intake and all-cause mortality in subgroups of age, gender, BMI, smoking, and dyslipidemia.

Subgroup	HR (95%CI)	*P*	HR (95%CI)	*P*	*P* for interaction
Subgroup I: Age	Age <65 years		Age ≥65 years		
Niacin intake	1.24 (0.99–1.56)	0.066	0.85 (0.74–0.98)	0.027	0.207
Niacin intake					
≤19mg	Ref		Ref		
19-33mg	1.22 (0.82–1.81)	0.322	0.98 (0.79–1.22)	0.853	
>33mg	1.39 (0.85–2.29)	0.190	0.68 (0.53–0.88)	0.003	
Subgroup II: Gender	Male		Female		
Niacin intake	0.89 (0.75–1.07)	0.216	0.90 (0.77–1.07)	0.231	0.179
Niacin intake					
≤19mg	Ref		Ref		
19-33mg	0.92 (0.74–1.15)	0.476	0.97 (0.75–1.25)	0.801	
>33mg	0.68 (0.51–0.92)	0.012	0.80 (0.58–1.09)	0.152	
Subgroup III: BMI	BMI <25kg/m^2^		BMI ≥25kg/m^2^		
Niacin intake	0.83 (0.65–1.06)	0.137	0.93 (0.81–1.07)	0.309	0.515
Niacin intake					
≤19mg	Ref		Ref		
19-33mg	1.16 (0.81–1.67)	0.423	0.93 (0.75–1.15)	0.501	
>33mg	0.70 (0.39–0.99)	0.049	0.80 (0.61–1.05)	0.103	
Subgroup IV: Smoking	No		Yes		
Niacin intake	0.95 (0.80–1.13)	0.577	0.89 (0.74–1.06)	0.199	0.164
Niacin intake					
≤19mg	Ref		Ref		
19-33mg	1.19 (0.90–1.55)	0.214	0.86 (0.67–1.12)	0.272	
>33mg	0.94 (0.67–1.31)	0.708	0.68 (0.49–0.94)	0.020	
Subgroup V: Dyslipidemia	No (n = 911)		No (n = 911)		
Niacin intake	1.14 (0.80–1.64)	0. 471	0.88 (0.78–0.99)	0.041	0.030
Niacin intake					
≤19mg	Ref		Ref		
19-33mg	0.94 (0.58–1.53)	0.799	0.99 (0.81–1.21)	0.937	
>33mg	1.28 (0.69–2.38)	0.425	0.71 (0.56–0.91)	0.007	
Subgroup VI: Hyperphosphatemia	No		Yes		
Niacin intake	0.88 (0.77–0.99)	0.055	1.03 (0.75–1.41)	0.862	0.882
Niacin intake					
≤19mg	Ref		Ref		
19-33mg	0.92 (0.76–1.12)	0.402	1.40 (0.97–2.01)	0.069	
>33mg	0.73 (0.58–0.91)	0.006	0.99 (0.54–1.80)	0.966	

HR: hazards ratio; CI: confidence interval; Ref: reference.

Subgroup I: adjusting gender, race, marital status, smoking, hemoglobin, phosphorus, white blood cell, diabetes, hypertension, dyslipidemia, cardiovascular disease, albumin creatinine ratio, eGFR, survey years, and total energy intake.

Subgroup II: adjusting age, race, marital status, smoking, hemoglobin, phosphorus, white blood cell, diabetes, hypertension, dyslipidemia, cardiovascular disease, albumin creatinine ratio, eGFR, survey years, and total energy intake.

Subgroup III: adjusting age, gender, race, marital status, smoking, hemoglobin, phosphorus, white blood cell, diabetes, hypertension, dyslipidemia, cardiovascular disease, albumin creatinine ratio, eGFR, survey years, and total energy intake.

Subgroup IV: adjusting age, gender, marital status, hemoglobin, phosphorus, white blood cell, diabetes, hypertension, dyslipidemia, cardiovascular disease, albumin creatinine ratio, eGFR, survey years, and total energy intake.

Subgroup V: adjusting age, gender, race, marital status, hemoglobin, phosphorus, white blood cell, diabetes, hypertension, cardiovascular disease, albumin creatinine ratio, eGFR, survey years, and total energy intake.

Subgroup VI: adjusting age, gender, race, marital status, hemoglobin, white blood cell, diabetes, hypertension, cardiovascular disease, albumin creatinine ratio, eGFR, survey years, and total energy intake.

## Discussion

Higher dietary niacin intake was related to lower all-cause mortality risk in CKD patients. The association was consistent across various subgroups, including age ≥65 years old, males, BMI <25 kg/m^2^, smoking, dyslipidemia, and non-hyperphosphatemia. Our findings suggested a potential beneficial role of niacin in the prognosis of CKD patients.

Comparing our findings with previous studies, limited studies have investigated the association of niacin with mortality in CKD patients. Anyway, our findings were consistent with studies that have explored the relationship between niacin intake and mortality [22, 23]. Pan et al. [22] reported higher dietary niacin intake was associated with a lower risk of all-cause mortality in nonalcoholic fatty liver disease patients. In diabetes, the relationship between niacin intake and reduced mortality was also observed [23]. While these studies did not specifically focus on CKD patients, they provided indirect support for the potential benefits of niacin intake on prognosis. In addition, the results suggested that niacin intake may be considered, particularly for CKD patients with dyslipidemia, where its lipid-modifying effects could be doubly beneficial.

The relationship between higher niacin intake and reduced all-cause mortality risk was observed in patients aged ≥65 years old. Advanced age often accompanies a higher burden of chronic conditions and physiological changes, including increased oxidative stress and inflammation, which are pathways through the protective effects of niacin’s antioxidant properties. The relationship between higher niacin intake and lower all-cause mortality risk in males with CKD is similar to Ying’s research [15]. Biological differences such as hormonal profiles and body composition could potentially modulate the impact of niacin on mortality risk in males versus females. Dyslipidemia represents a common complication in CKD, contributing to accelerated cardiovascular disease and mortality [24]. Niacin’s lipid-modifying effects may have a potential impact on mitigating mortality risk in this population. In CKD patients with BMI< 25 kg/m^2^, nutritional status and metabolic demands may differ, potentially affecting niacin utilization and efficacy. Similarly, smoking behavior introduces additional oxidative stress and inflammatory pathways, which could interact with niacin’s mechanisms of action. Dyslipidemia might influence how niacin affects mortality risk in CKD patients. Niacin could improve lipid profiles by increasing HDL-C, LDL-C, and TG. In patients with dyslipidemia, who already have altered lipid metabolism, niacin’s effect on lipid levels could be more pronounced, potentially leading to benefits in reducing cardiovascular events and, consequently, all-cause mortality.

The mechanisms underlying the observed association between niacin intake and reduced mortality risk in CKD patients were unclear. However, several potential pathways may contribute to the association. One possible mechanism is niacin’s critical role in cellular metabolism and energy production. It acts as a precursor to nicotinamide adenine dinucleotide (NAD+) and its phosphorylated form (NADP+), essential coenzymes involved in numerous biochemical reactions, including oxidative phosphorylation and ATP synthesis [25]. These processes are vital for cellular function and survival, particularly in tissues with high energy demands such as the kidney.

Another potential mechanism is niacin’s antioxidant and anti-inflammatory properties. CKD is associated with chronic inflammation and increased oxidative stress, both of which contribute to the progression of CVD and mortality in CKD patients [26, 27]. Niacin has been shown to possess antioxidant properties by reducing reactive oxygen species production and enhancing the activity of antioxidant enzymes [28]. Furthermore, niacin can modulate the expression of inflammatory markers, such as C-reactive protein and interleukin-6, thereby attenuating inflammation [9, 29]. By reducing oxidative stress and inflammation, niacin may help mitigate the adverse effects of these processes on cardiovascular health and mortality in CKD patients. Additionally, niacin has vasodilatory effects, primarily mediated by the activation of G-protein-coupled receptor 109A (GPR109A), also known as niacin receptor 1 [30]. Activation of GPR109A promotes the release of endothelium-derived relaxing factors, such as nitric oxide, leading to vasodilation and improved endothelial function [31]. The vasodilatory effect may contribute to the improvement of blood flow and oxygen supply to vital organs, thereby reducing the risk of CVD events and mortality.

The association between higher dietary niacin intake and lower odds of all-cause mortality suggests that niacin supplementation or dietary modifications could be considered as a potential adjunct therapy in CKD management. Although current guidelines do not specifically recommend niacin intake for CKD patients, our study supports the notion that adequate niacin intake may be beneficial to prognosis in CKD patients. However, further prospective studies and randomized controlled trials are warranted to validate our findings and establish optimal niacin supplementation strategies.

Several limitations exist in our study. First, the retrospective nature of the cohort study design limits the ability to establish causality between niacin intake and all-cause mortality in CKD patients. Additionally, dietary niacin intake was assessed via 24-hour dietary recall interviews, which are subject to recall bias. Furthermore, our study did not account for potential confounding factors which were not included in the NHANES database. As they may influence the association of dietary niacin intake with all-cause mortality.

### Conclusion

Higher dietary niacin intake was associated with lower odds of all-cause mortality in CKD patients. The association was also observed across various subgroups, including age ≥65 years old, male, BMI <25 kg/m^2^, smoking, dyslipidemia, and non-hyperphosphatemia. Our findings suggest the potential role of dietary niacin as part of a comprehensive dietary strategy in CKD management. Further study is warranted to confirm the mechanisms behind the association and to determine the clinical applicability of niacin supplementation in CKD patients.

## Supporting information

S1 Table The distribution of missing values. (DOCX)

S2 Table Sensitivity analysis before and after interpolation of missing values. (DOCX)

S1 File (DOCX)
