# Supplementary material for: Association of dietary niacin intake with all-cause mortality in chronic kidney disease: A retrospective cohort study of NHANES
Source: PLoS One. 2025 Feb 7;20(2):e0313398. doi: 10.1371/journal.pone.0313398 (PMC11805444; doi:10.1371/journal.pone.0313398)
Supplement: S1 Table — (DOCX) [file pone.0313398.s001.docx]

**Supplementary Table 1.** The distribution of missing values

| Variables | n (%) |
| --- | --- |
| Education | 137 (2.94%) |
| Marital status | 130 (2.79%) |
| Poverty-to-income ratio | 441 (9.47%) |
| Smoking | 80 (1.72%) |
| Drinking | 398 (8.54%) |
| BMI | 109 (2.34%) |
| Uric acid | 252 (5.41%) |
| WBC | 178 (3.82%) |
| Phosphorus | 250 (5.37%) |
| Hemoglobin | 178 (3.82%) |
| Depression | 366 (7.86%) |
