# Supplementary material for: Association of dietary niacin intake with all-cause mortality in chronic kidney disease: A retrospective cohort study of NHANES
Source: PLoS One. 2025 Feb 7;20(2):e0313398. doi: 10.1371/journal.pone.0313398 (PMC11805444; doi:10.1371/journal.pone.0313398)
Supplement: S1 File — (DOCX) [file pone.0313398.s003.docx]

**Supplementary Table 1.** The distribution of missing values

| Variables | n (%) |
| --- | --- |
| Education | 137 (2.94%) |
| Marital status | 130 (2.79%) |
| Poverty-to-income ratio | 441 (9.47%) |
| Smoking | 80 (1.72%) |
| Drinking | 398 (8.54%) |
| BMI | 109 (2.34%) |
| Uric acid | 252 (5.41%) |
| WBC | 178 (3.82%) |
| Phosphorus | 250 (5.37%) |
| Hemoglobin | 178 (3.82%) |
| Depression | 366 (7.86%) |

**Supplementary Table 2.** Sensitivity analysis before and after interpolation of missing values

| Variables | Before interpolation | After interpolation | Statistics | P |
| --- | --- | --- | --- | --- |
| Education, n (%) |  |  | χ^2^=3.44 | 0.179 |
| Less than high school | 1446 (22.85) | 1488 (22.77) |  |  |
| High school | 1100 (26.40) | 1136 (26.70) |  |  |
| College and above | 1976 (50.75) | 2035 (50.53) |  |  |
| Marital status, n (%) |  |  | χ^2^=3.86 | 0.145 |
| Married | 2170 (50.21) | 2228 (49.87) |  |  |
| Never married | 534 (13.07) | 553 (13.10) |  |  |
| Others | 1825 (36.72) | 1878 (37.03) |  |  |
| PIR, n (%) |  |  | χ^2^=1.12 | 0.290 |
| <1.0 | 1055 (19.06) | 1172 (19.29) |  |  |
| ≥1.0 | 3163 (80.94) | 3487 (80.71) |  |  |
| Smoking, n (%) |  |  | χ^2^=0.64 | 0.424 |
| No | 2331 (51.04) | 2377 (51.12) |  |  |
| Yes | 2248 (48.96) | 2282 (48.88) |  |  |
| Drinking, n (%) |  |  | χ^2^=3.02 | 0.082 |
| No | 1536 (32.53) | 1684 (32.95) |  |  |
| Yes | 2725 (67.47) | 2975 (67.05) |  |  |
| BMI | 30.33 (0.19) | 30.32 (0.19) | t=0.33 | 0.742 |
| Uric acid | 5.90 (0.04) | 5.91 (0.03) | t=-1.75 | 0.083 |
| WBC | 7.69 (0.06) | 7.69 (0.06) | t=0.07 | 0.947 |
| Phosphorus | 3.76 (0.01) | 3.76 (0.01) | t=-0.85 | 0.395 |
| Hemoglobin | 13.74 (0.05) | 13.75 (0.05) | t=-1.31 | 0.195 |
| Depression, n (%) |  |  | χ^2^=0.01 | 0.907 |
| No | 3794 (89.54) | 4119 (89.52) |  |  |
| Yes | 499 (10.46) | 540 (10.48) |  |  |

t: t test; χ^2^: chi-square tests.
